# On the Value of Crystal Gold in Dentistry

**Published:** 1892-02

**Authors:** N. W. Williams

**Affiliations:** Nice, France


### ON THE VALUE OF CRYSTAL GOLD IN DENTISTRY.[1]

BY N. W. WILLIAMS, D.D.S., NICE, FRANCE.

Crystal gold was first brought to the notice of the profession as early as 1855 or 1856. In the winter of 1856 or 1857 I was attending my first course in the Ohio Dental College, at Cincinnati, and Professors George Watt and J. Taft had invented a form of crystal gold which they exhibited before the class, filling a number of teeth to demonstrate its superiority over gold-foil. Professor Taft built up for me before the class a superior molar, broken down on three sides to the gum, with this new gold, and it was considered a wonderful operation, as this was before the days of the advent of the rubber dam and the mallet, and all of the other aids to operative dentistry which we now possess. This filling was one of the wonders of the day, and it had to be exhibited at all of the associations for years.

This form of gold was made after what is called the mercury process, which consists in dissolving pure gold in one part of pure nitric acid to three parts of pure hydrochloric acid, and the gold precipitated by slowly adding a strong solution of sulphate of iron and letting it stand undisturbed for about twenty-four hours, when the gold may be found in the bottom of the vessel in a brown powder. The solution is carefully poured off and the gold is poured onto a flat dish and pure sulphuric acid added, sufficient to cover the gold, and allowed to stand for a few hours to get rid of any iron which may be present. Then the gold is washed with several workings of distilled boiling water until no taste of acid remains. It is then allowed to dry until all moisture disappears. It is placed with pure mercury, a little at a time, in a wedgewood mortar and ground together until it is a thick paste, when it is again washed with hot distilled water until the water remains clear. It is then placed in an evaporating dish, a steam bath, and dilute nitric acid added to cover the mass, and watched closely until all of the mercury has been driven off, which leaves the gold in crystal form. This is washed with distilled hot water until no taste of acid remains. Then the gold must be placed on a soapstone slab and put into the fire and slowly heated to redness, which drives off any impurities and anneals it, and it is then fit for use. The process being

[1] Read before the American Dental Society of Europe at Heidelburg, August 4, 1891.

somewhat difficult and complicated and attended with some danger to the health, the inventors were never able to make it in sufficient quantities for sale, and the profession were deprived of a very valuable aid in operative dentistry.

Becoming associated in practice with Professor Watt in 1865, we continued to make it for our own use during the seven years of our association, and I have never, before or since, filled teeth with such satisfaction to myself and patients. After coming abroad to practise, I did not have the same facilities for its manufacture, and I was forced to resort to the hard work of making fillings with gold-foil.

The crystal gold, known under the name of A. J. Watt's crystal or sponge gold, was brought out about the same time as that of Watt and Taft. At first it was not a success, as complaints were made that it discolored in the mouth, and did not make a perfect filling at the margins. This may have been due in part to bad manipulation, for, being very spongy, one was inclined to use it in too large pieces, and it then would harden under the instrument before it was condensed throughout the mass. The makers of this gold have steadily improved it until now it is a very valuable gold in saving teeth.

Of late I have been using it *in connection with gold-foil*, and I find they work well together, and are a great help one to the other. My method consists in placing some soft gold-foil, either in the form of rope or cylinders, against the wall or part of the cavity where I am to begin my filling, and then with the pliers take up a piece of the crystal gold sufficient to cover the foil, and hold it in the flame of the spirit lamp until it is heated to redness, then place it against the soft foil, and with the mallet drive it home at the same time with an instrument in the left hand, holding the soft gold in position until the first piece is perfectly condensed. Then I proceed in the same manner until all of the walls are covered or lined. In using the soft foil against the walls it takes the form better than if it were annealed, and makes a better filling, while at the same time, the crystal gold being hot, it sufficiently anneals the soft foil at point of contact to cause the two kinds of gold to perfectly unite together. After all the walls are covered, and the soft foil having been allowed to come up to and cover the margins, I proceed with the filling, using alternately the crystal gold and foil, but annealing or heating each piece of foil, as well as the crystal gold. When first beginning this method of filling I used little balls of loose cotton-wool, which I placed against the soft foil, and with the plugger and the mallet would drive the soft gold against the walls. This leaves the cavity beautifully lined with gold. But I found the crystal gold answered the same purpose, and the lining was not so apt to become displaced. In very deep cavities and under-cuts the cotton I find very useful in getting the gold where I want it. I always try to have the last layers of gold in the filling to lie with the crystal gold and condensed with instruments with fine serrations, as there is no gold yet discovered that makes so hard and fine a finish as the crystal gold. I feel quite confident that any operator, after a few trials, will be convinced that crystal gold has a value in dentistry that is not generally appreciated.

